# µFBI: A Microfluidic Bead-Based Immunoassay for Multiplexed Detection of Proteins from a µL Sample Volume

**DOI:** 10.1371/journal.pone.0013125

**Published:** 2010-10-01

**Authors:** Xiaobo Yu, Michael Hartmann, Quan Wang, Oliver Poetz, Nicole Schneiderhan-Marra, Dieter Stoll, Cornelia Kazmaier, Thomas O. Joos

**Affiliations:** 1 Natural and Medical Sciences Institute (NMI), University of Tübingen, Reutlingen, Germany; 2 Department of Analytical Chemistry, School of Chemical Science and Engineering, Royal Institute of Technology, Stockholm, Sweden; 3 Experimentelle and Diagnostische Immunologie (EDI) GmbH, Reutlingen, Germany; 4 Hochschule Albstadt-Sigmaringen, Sigmaringen, Germany; Deutsches Krebsforschungszentrum, Germany

## Abstract

**Background:**

Over the last ten years, miniaturized multiplexed immunoassays have become robust, reliable research tools that enable researchers to simultaneously determine a multitude of parameters. Among the numerous analytical protein arrays available, bead-based assay systems have evolved into a key technology that enables the quantitative protein profiling of biological samples whilst requiring only a minimal amount of sample material.

**Methodology/Principal Findings:**

A microfluidic bead-based immunoassay, µFBI, was developed to perform bead-based multiplexed sandwich immunoassays in a capillary. This setup allows the simultaneous detection of several parameters and only requires 200 ng of tissue lysate in a 1 µL assay volume. In addition, only 1 µL of detection antibodies and 1 µL of the reporter molecule Streptavidin-Phycoerythrin were required. The µFBI was used to compare the expression of seven receptor tyrosine kinases and their degree of tyrosine phosphorylation in breast cancer tissue and in normal tissue lysates. The total amount of HER-2, as well the degree of tyrosine phosphorylation was much higher in breast cancer tissue than in normal tissue. µFBI and a standard bead-based assay led to identical protein expression data. Moreover, it was possible to reduce the quantity of sample material required by a factor of 100 and the quantity of reagents by a factor of 30.

**Conclusions/Significance:**

The µFBI, microfluidic bead-based immunoassay, allows the analysis of multiple parameters from a very small amount of sample material, such as tumor biopsies or tissue sections.

## Introduction

Over the last ten years, protein microarray technologies have progressed to become effective multiplex analysis tools for assessing the expression and function of proteins from a small amount of sample material [1]–[6]. Microarrays are solid phase-based assay systems consisting of an array of miniaturized test sites, in which many tests can be performed in parallel. Planar protein microarrays use capture molecules that are immobilized in microspots of rows and columns, making it possible to analyze a large number of parameters simultaneously [7]–[10]. In analogy to the spatial separation employed by planar microarrays, bead-based systems employ color-coded or size-coded microspheres to identify different immunoassays. Different color-coded microspheres are coated with different capture antibodies and incubated with the samples of interest. A secondary detection antibody and a reporter molecule are used to visualize the captured analytes. The individual bead types are identified in a flow cytometer and the number of bead-captured analytes is determined. Bead-based systems have emerged as very interesting alternatives to planar microarrays, especially in focused analyses where the number of parameters to be analyzed simultaneously is relatively small and the number of samples to be analyzed is quite high [11]. Luminex's xMAP technology is the most advanced bead-based technology currently available; it involves a flow cytometry system that can handle 96-well microtiter plates and is equipped with advanced digital signal processing hardware and software. Luminex microspheres are 5.6 µm in diameter and stained with different proportions of a red and an infrared dye, which results in 100 distinct color-coded beads. The beads enable researchers to screen up to 100 parameters in a single experiment. Such bead-based assay systems are flexible, robust, and, in contrast to planar microarrays, more advanced in terms of automation [12]. There is a growing list of commercially available, ready-to-use, multiplexed bead-based assays for the quantification of cytokines and cell-signaling molecules and the analysis of kinase activity (www.biochipnet.de, Biochipnet).

The information obtained from multiplexed assays helps in the detection of molecular events in the early stages of cancer progression and in the early diagnosis of cancer. As early-stage tumor sample size is usually small, therefore it is only possible to obtain small amounts of material, for example, fine needle aspiration [13]–[15]. The identification of changes in protein expression in very small samples is especially challenging since only a limited number of assays can be performed using conventional approaches. It goes without saying that the potential of genomic and proteomic technologies can only be fully exploited if they can be applied to minute amounts of biological material [16]–[19].

Multiplexed immunoassays based on protein microarray platforms have been broadly employed in the discovery and validation of disease-associated biomarkers as well as in clinical diagnostics research [20]–[25]. However, there is still a great need for integrated microfluidic test devices which would ideally perform multiplexed immunoassays in a controlled environment whilst using only small amounts of sample material, like fine needle biopsies or microdissected tissue sections. The present study presents a microfluidic, bead-based immunoassay (µFBI) approach for the multiplexed detection of proteins involving a capillary to control the application of minute amounts of liquid. Performing an immunoassay inside a capillary requires only 200 ng tissue lysate present in 1 µL sample volume, 1 µL detection antibody solution and 1 µL of reporter molecule streptavidin-phycoerythrin. This corresponds to a 100-fold and 30-fold reduction in sample and reagents compared to standard bead-based immunoassays. The present paper describes the setup of the microfluidic bead-based immunoassay and demonstrates the performance of the µFBI by analyzing the expression of receptor tyrosine kinases in lysates from breast cancer and normal tissue.

## Results and Discussion

Multiplexed immunoassay can be performed in a capillary requiring only a minute amount of sample material. Phillips et al. (2007) analyzed the expression of twelve cytokines in dissected tissue lysates. The cytokines were captured by a mixture of immobilized capture antibodies, and subsequently labeled with a flurophore. Captured and labeled cytokines were separated by electrophoresis and the quantification of individual cytokine peaks was performed [26]–[28]. For this approach high quality capture antibodies are required revealing high-affinity and high-specificity [27]. O'Neill et al. reported a high-resolution capillary isoelectric focusing technology which resolved isoforms and individual phosphorylation forms of target analytes. The separated proteins and their isoforms were immobilized by a photochemical capture method in the capillary, and subsequently visualized with specific antibodies using a chemiluminescence based readout [29]–[30]. Good sensitivity and resolution of protein isoforms could be achieved with this method. However, the ability of multiplexing of this approach is limited and only a few numbers of analytes can be analyzed simultaneously. As an alternative approach, we applied multiplexed sandwich immunoassay technology and performed a micro fluidic bead-based immunoassay –µFBI – in a capillary.

### Optimization of relevant parameters for microfluidic bead-based immunoassay – µFBI

**Figure 1:**
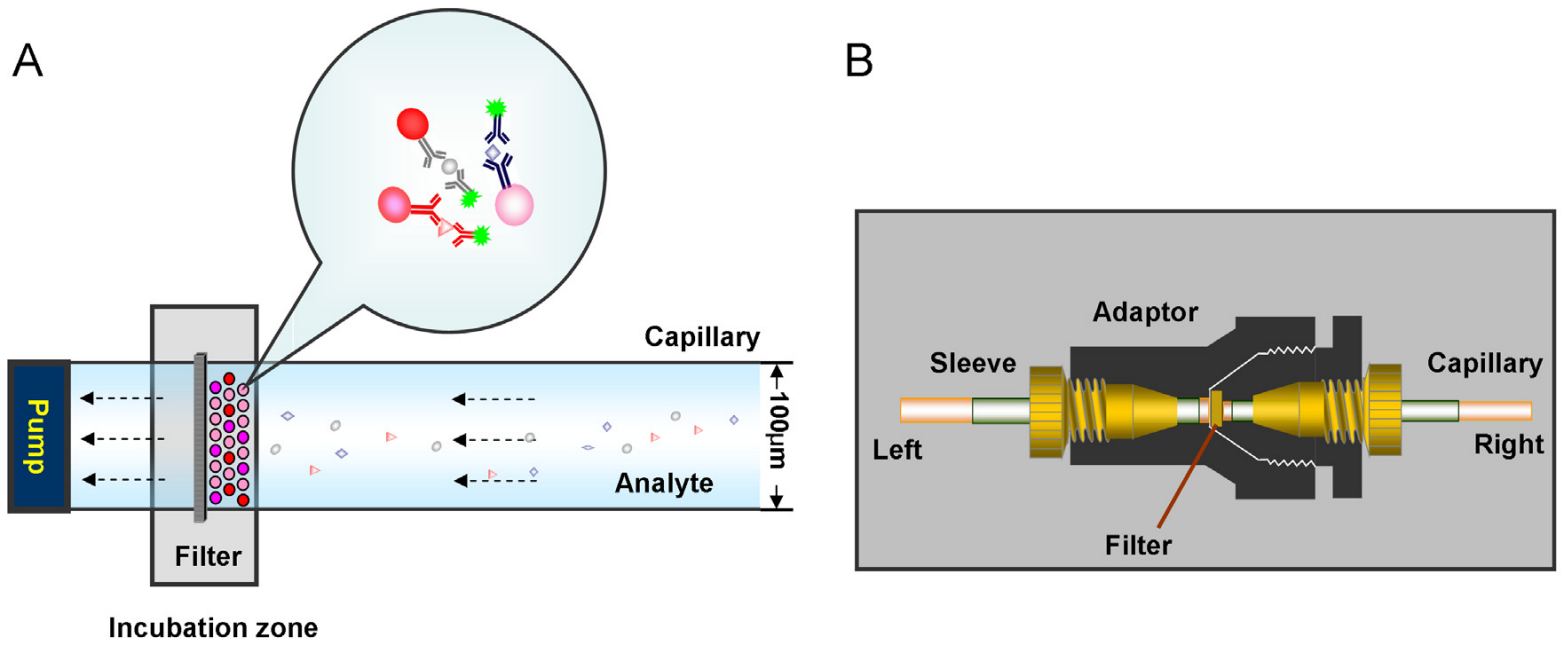
Schematic illustration of microfluidic bead-based immunoassay (µFBI). (A) The assay is performed in a capillary by controlling the solution flow into or out of the capillary with a syringe pump. A filter (pore diameter = 0.5 µm) is used to keep the antibody-coated beads at the incubation site to capture target analytes and to react with detection reagents. After incubation, the beads are pumped out and submitted to a Luminex 100 IS instrument for fluorescent signal readout. (B) The structure of the incubation zone consists of a filter and an adaptor to connect two fused silica-capillaries at both sides. The left capillary is connected to a syringe pump and the right capillary is connected to a sample solution reservoir.

The schematic set up for µFBI is shown in Figure 1A. It consists of a syringe pump, a syringe, an incubation zone and two capillaries. The incubation zone is comprised of a filter and an adapter to connect two capillaries at both sides. The left capillary is connected to the syringe pump and the right capillary is connected to the sample solution reservoir (Figure 1B). The µFBI can be performed by controlling the solution flow in and out of the capillary with the syringe pump.

**Figure 2:**
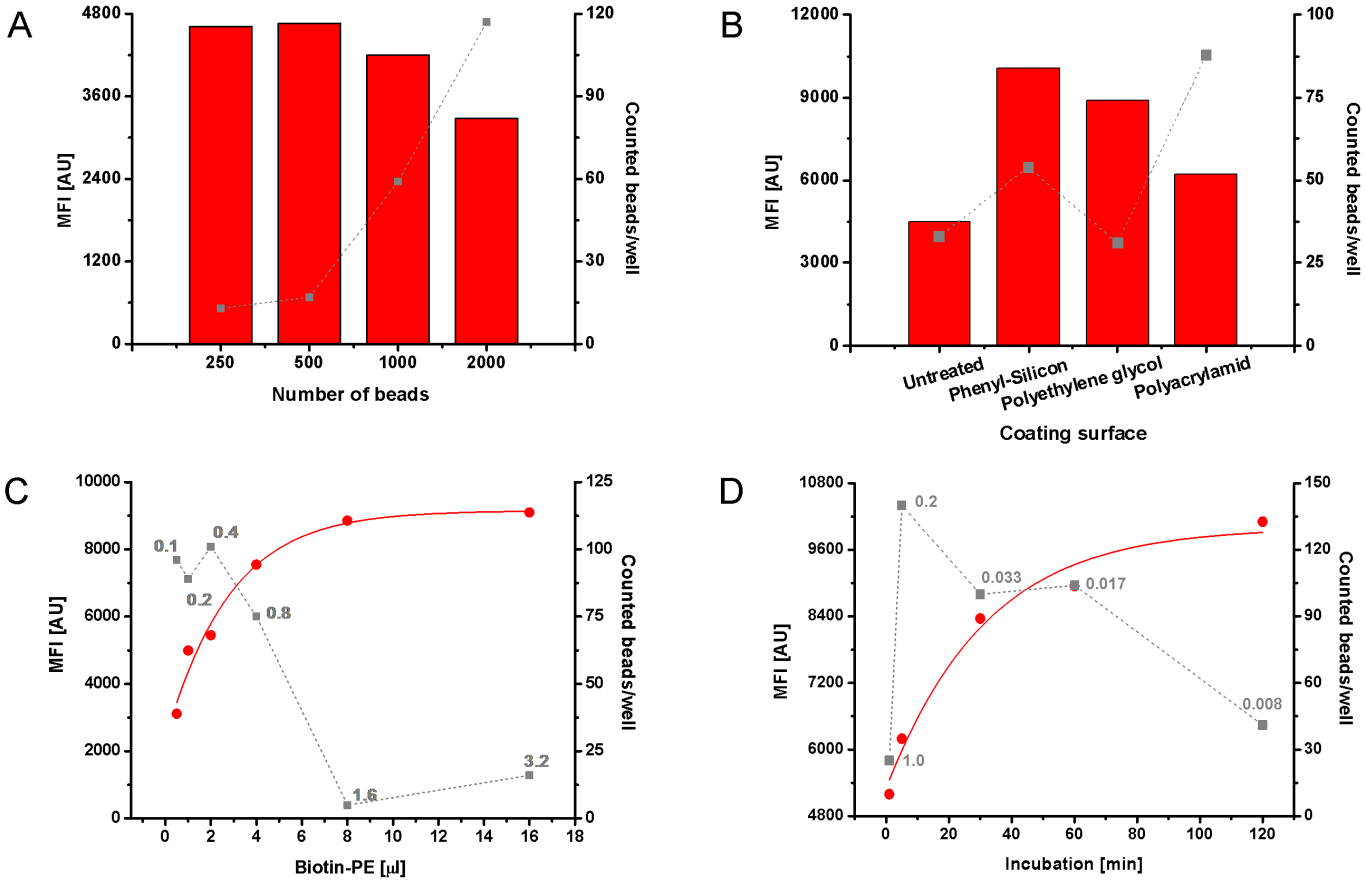
Optimization of µFBI-relevant parameters. (A – D) denote the optimization of bead number, capillary coating surface, analyte volume, and incubation time, respectively. In the experiment, anti-biotin antibody-coated beads were incubated in the capillary with biotin-PE (100 ng/ml). The resulting beads were then pumped out and diluted in 100 µL with assay diluent and analyzed with the Luminex 100 IS system using fluorescent detection. The numbers in gray given in C and D represent the flow rate of the sample solution. The MFI value (column for A and B, circle for C and D) was read out and the recovered beads (square) were counted with the Luminex 100 IS system.

The relevant parameters of the µFBI were optimized using an artificial capture assay consisting of an immobilized anti-biotin antibody and biotinylated PE as the analyte. The optimal number of antibody coupled beads was defined in a first experiment. 250, 500, 1000, or 2000 beads were pumped into the capillary and incubated for 5 min with Biotin-PE (100 ng/ml). The median fluorescent intensity (MFI) and the number of recovered beads were analyzed (Figure 2A). The MFI value reflects the amount of captured Biotin-PE and the number of recovered beads correlates to the robustness of the MFI signal. At least 35 recovered beads should be obtained to achieve a robust MFI value due to statistical requirements for the use of median signals. The number of recovered beads increased with an increasing starting amount of beads. Using 1000 and 2000 beads in the assay more than 50 beads were counted resulting in a robust MFI signal. Therefore, 1000 beads for each bead type were used in further experiments.

Next, the influence of the coating surface of capillary was investigated. Capillaries with four different physical surface properties (untreated, phenyl-silicon, polyethylene glycol and polyacrylamid) were tested for their performance of the µFBI assay (Figure 2B). The highest MFI values and a sufficient number of recovered beads above 50 was obtained using the capillary with a hydrophobic phenyl-silicon coating surface. This might be due to the very low flow rate, which may not transport all the beads through the capillary.

Thirdly, the optimal assay volume (0.5, 1, 2, 4, 8 and 16 µl) was determined using the Streptavidin - Biotin-PE assay. 1000 anti-biotin antibody coated beads were incubated with Biotin-PE (100 ng/ml) for 5 min by changing the flow rate from 0.1, 0.2, 0.4, 0.8, 1.6 up to 3.2 µl/min. The generated MFI and the number of recovered beads are given in Figure 2C. The data revealed that the MFI value was enhanced steadily when sample volume was increased from 0.5 to 16 µl. However, the number of recovered beads decreased below 50 when the volume exceeded 4 µl and the flow rate was above 0.8 µl/min, respectively.

The optimal incubation time was determined using the Streptavidin - Biotin-PE assay. The different incubation periods (1, 5, 30, 60 and 120 min) of the anti-biotin antibody coated beads with 1 µL Biotin-PE (100 ng/ml) were achieved by adjusting the flow rate from 1 µl/min, down to 0.008 µl/min. The results revealed that the MFI value increased with longer incubation times whereas the number of recovered beads decreased below 50 when the incubation time was extended above 60 min (Figure 2D). These data revealed that high flow rates result in shorter incubation times but with the downside of a poor bead recovery. An increase in sensitivity of the µFBI could be achieved using larger sample volumes while keeping the incubation time constant (Figure 2C), or increase the incubation time while keeping the sample volume constant (Figure 2D).

**Figure 3:**
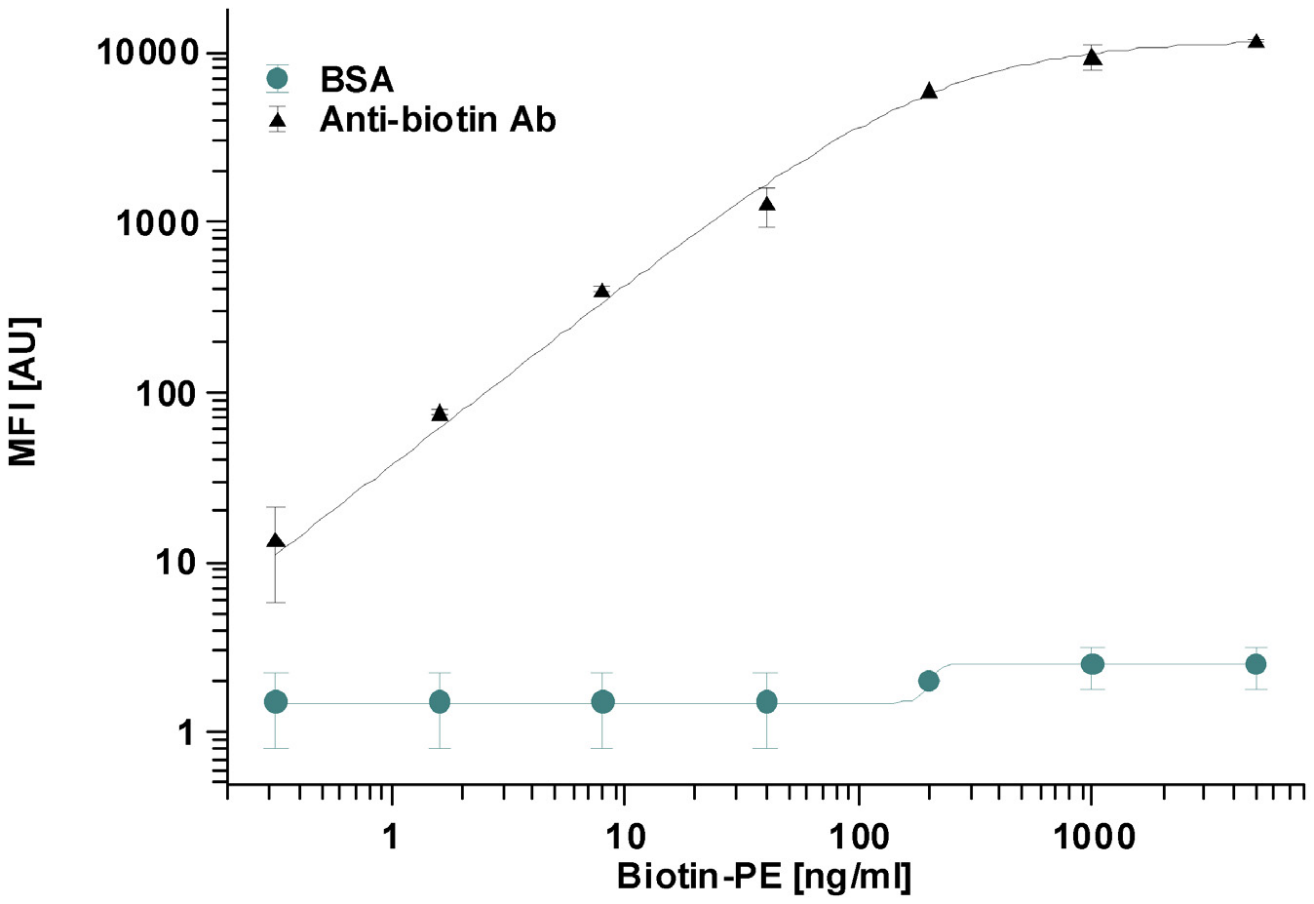
Quantification of Biotin-Phycoerythrin using the µFBI assay. 1,000 anti-biotin antibody-coated beads were incubated with different concentrations of biotin-phycoerythrin (1 µl) for 5 min, respectively. A BSA-coated bead served as a control. The MFI generated with the anti-biotin antibody (triangle) or the BSA-coated beads (circle) are shown.

Finally, the performance of µFBI assay was evaluated by the quantitative analysis of different biotin-PE concentrations. Anti-biotin antibody coated beads were incubated with 1 µl of Biotin-PE using concentrations ranging from 0 to 5000 ng/ml for 5 min, respectively. As a negative control BSA coated beads of a differently color-coded bead type were used in the same assay. The data are shown in Figure 3. At the lowest Biotin-PE concentration of 0.32 ng/ml, the MFI of signal to background was still 14/3. The dynamic range of the assay was from 0.32 to 500 ng/ml. Unspecific signals observed on the BSA coated beads increased slightly at Biotin-PE concentrations above 100 ng/ml.

These sets of experiments allowed to define the number of beads (1000), the surface coating (phenyl-silicon), the analyte volume (1 µL), incubation time (60 min) and quantitation ability of the µFBI assay. In a next step, the µFBI was applied to the analysis of Receptor Tyrosine Kinases in tissue lysates.

### Multiplexed detection of seven Receptor Tyrosine Kinases in breast cancer tissue

A commercially available bead-based sandwich immunoassay - WideScreenTM Receptor Tyrosine Kinase Assay (RTK) Kit - was selected to evaluate the performance of the µFBI. RTKs are critical regulators of numerous cell signaling pathways. Ligand binding to the extracellular domain of transmembrane RTKs triggers receptor dimerization and autophosphorylation of an intracellular kinase domain. This event ultimately triggers activation of downstream pathway proteins via phosphotyrosine-SH2 domain interactions. RTKs have been shown to be not only key regulators of normal cellular processes but also to have a critical role in the development and progression of many types of cancer [31]–[33]. For seven RTKs - Epidermal Growth Factor Receptor (EGFR), Insulin-like Growth Factor 1 Receptor (IGF-1R), Hepatocyte Growth Factor Receptor (HGFR), Platelet-Derived Growth Factor Receptor beta (PDGFRβ), Human Epidermal Growth Factor Receptor 2 (HER-2), Vascular Endothelial Growth Factor Receptor 2 (VEGFR2) and Tyrosine Kinase with Immunoglobulin and EGF Repeats 2 (Tie-2) - the total amount and the phosphorylation levels were measured in a breast cancer and a normal tissue lysate using the standard bead-based sandwich immunoassay protocol and the µFBI.

**Figure 4:**
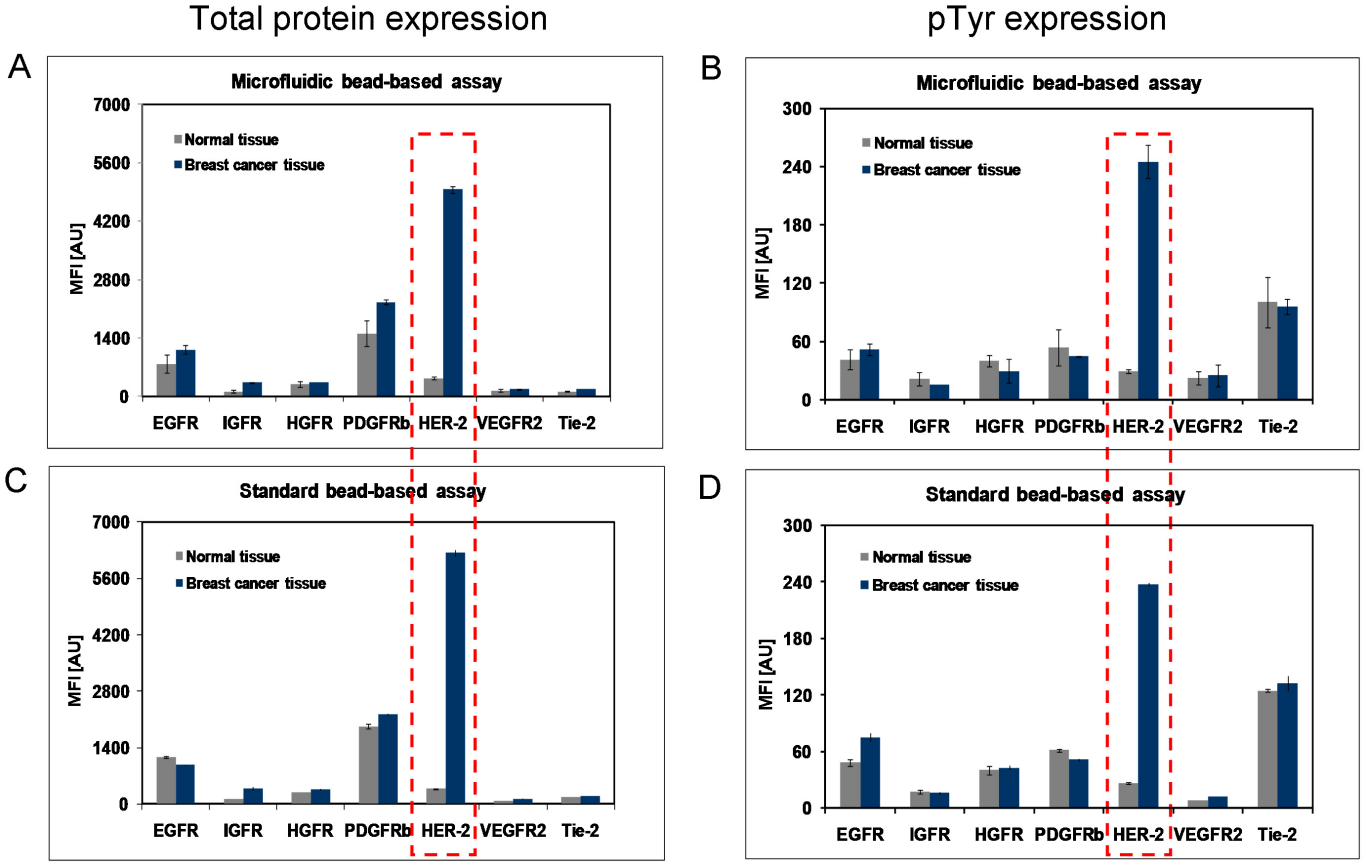
Protein expression analysis using the µFBI. Total protein expression and phosphotyrosine-specific expression of seven RTKs were determined by µFBI (A and B) and a normal bead-based assay (C and D). For µFBI, seven mixed capture antibody-coated beads (1,000 beads per analyte) were incubated with tissue lysates (1 µl) for 60 min, mixed biotinylated detection antibodies (1 µl) for 60 min and Strep-PE (1 µl) for 45 min. The MFI value was obtained with a Luminex 100 IS system.

Considering the relationship between the assay sensitivity and incubation time as well as influence of flow rate (Figure 2D), the capture antibody coated beads were incubated for 60 min with the tissue lysates, for 60 min with biotinylated detection antibody mix and subsequently for 45 min with the reporter molecule Streptavidin-PE (each 1 µL). In parallel, a standard bead-based assay was performed according to the manufacturer's instruction. Signal intensities representing the RTK concentrations (Fig. 4A, C) and their degree in Tyrosine phosphorylation (Fig. 4B, D) correlated very well between the µFBI and the standard bead-based assay. No significant differences of the fluorescent intensities of the different analytes between both methods were observed (Figure 4). Both methods revealed for HER-2 that both the total amount and the degree of Tyrosine phosphorylation were significantly higher in breast cancer tissue compared to normal tissue. However, the µFBI only required 200 ng of tissue lysates, compared to 20 µg in the standard bead-based assay. For the detection antibody mix and the reporter solution Streptavidin-PE the µFBI required 30-fold less material (Table 1).

**Table 1:** Comparison of µFBI and standard bead-based assay.

Procedure	Micro fluidic bead-based assay		Standard bead-based assay		Fold reduction
	Time	Usage per capillary	Time	Usage per well	
Pre-wet	10 min	20 µL	5 min	100 µL	
Beads		1 µL, 1000beads/analyte		30 µL, 2000beads/analyte	
Vacuum	-	-			
Sample	60 min	1 µL, 200 ug/ml	60 min	100 µL, 200 ug/ml	**100**×
3×wash	-	-			
Detection antibody	60 min	1 µL, 1×	60 min	30 µL, 1×	**30**×
3×wash	-	-			
Strep-PE	60 min	1 µL, 1×	60 min	30 µL, 1×	**30**×
3×wash	-	-			
Recover beads	3 min	5 µL	-	-	
Measurement	80 µL, 80 s, Gate: 7500–15000		80 µL, 80 s, Gate: 7500–15000		

-: without this procedure.

**Figure 5:**
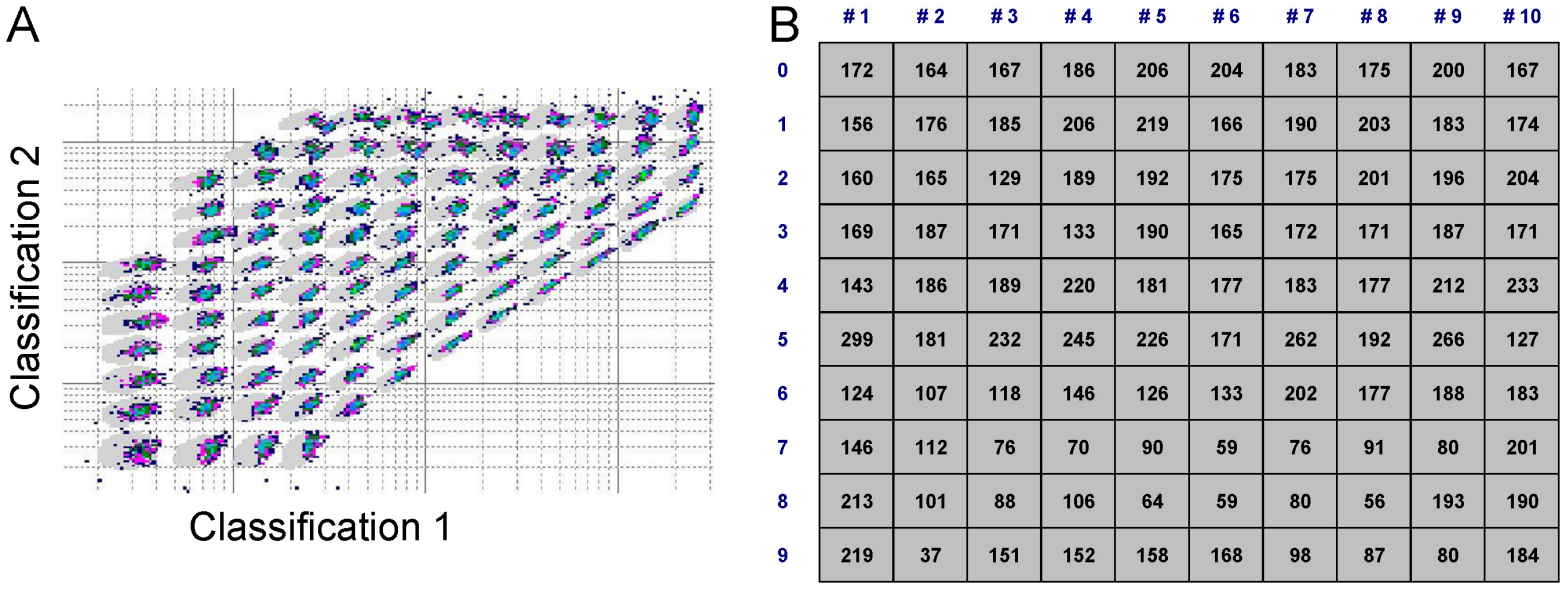
Simulation of a 100-plex assay. 800 beads of each bead type were incubated in the capillary for 1 h. Beads were pumped out and counted with a Luminex-100 instrument. (A) Counted beads of the 100 different color-coded bead types which can be spectrally distinguished by their colour code. (B) number of counted beads for each bead type.

Multiplexing of beads in the µFBI is not limited due to the very low sample volume of only 1 µL. In such a volume 100 different bead types can be incubated simultaneously and subsequently analyzed in a Luminex instrument. A set of 100 different bead types were incubated into the capillary for 1 h mimicking real incubation conditions. After this incubation time beads were pumped out of the capillary and counted in a Luminex 100 instrument. For each bead type a sufficient number of beads above 35 were counted which are sufficient to fulfill statistical requirements, when measuring MFIs (Figure 5).

Several aspects have to be considered to perform a successful µFBI experiment. Firstly, bubbles in the capillary have to be avoided, as bubbles severely influence the accuracy of bead numbers or sample volume during the assay. According to our experience, this problem could be significantly minimized by tightly sealing the connection between the microfilter and capillary and a carefully operation. In addition the dead volume of beads or sample has to be controlled. In our experiment, the capillaries were washed thoroughly with washing buffer in between consecutive assay runs. After extensive washing no residual beads could be observed, which allowed us to reuse the setup. In the here presented experimental setup two capillaries were used allowing to perform two assays in parallel. To increase sample throughput the number of capillaries can be increased as described recently by Bainer et al. [34]. Bainer et al. adapted a CE based separation of proteins to the microtiter plate format and used 96 capillaries in parallel. The integration of a flow cytometry based read-out system in such a set up could extend the µFBI concept to a compact and automatable system which would allow to process large number of samples in parallel and simultaneously screen for several dozens of analytes in a single experiment [35].

## Materials and Methods

### Beads coupling

2.5×10^6^ carboxylated colour coded beads with an diameter of 5.6 µm were obtained from Luminex, Inc. (Austin, TX, USA), and were coupled with 25 µg (100 µg/ml) Bovine serum albumin (BSA, Sigma, Inc., MO, USA)) or 25 µg (100 µg/ml) anti-biotin antibody (Sigma, Inc., MO, USA) according to the manufacturer's protocol. The coupled beads were stored at 4°C in Roche buffer (Roche Diagnostics GmbH, Mannheim, Germany) with 0.05% sodium azide. Coupling controls were used to assess the immobilization efficiency by staining 1500 of each type of microspheres with 0, 0.1, 0.3, 1 and 3 ug/ml R-phycoerythrin conjugated goat anti-mouse IgG (Jackson ImmunoResearch Laboratories, Inc., Suffolk, UK).

### µFBI setup

The µFBI set up consists of a syringe pump, incubation zone and two capillaries (Figure 1A). The KDS 200 syringe pump (KD Scientific, Inc., Holliston, MA, USA) holds two syringes (25 µL, Model 702, Innovative Labor Systeme GmbH, Stützerbach, Germany) which can perform the assay in duplicate. The incubation zone consists of a microfilter (M-520, 0.5 µm porosity, PEEK, IDEX Health & Science, Wertheim-Mondfeld, Germany) and an adapter (Upchurch Scientific Oak Harbor, WA, USA) to connect two fused silica-capillaries (Chromatographie Service GmbH, Berlin, Germany). The left capillary (diameter = 200 µm) is connected to a syringe and the right capillary (diameter = 100 µm) is connected to a sample solution (Figure 1B).

### Microfluidic bead-based assay

The WideScreen™ Receptor Tyrosine Kinase Assay Kit was obtained from EMD Chemicals Inc. (Gibbstown, NJ, USA). This kit enables the quantification of a set of key RTKs, including EGFR, IGFR, HGFR, PDGFRb, HER-2, VEGFR2, and Tie-2. Breast cancer tissue and normal tissue were kindly provided by Georg Sauer and Helmut Deissler, University hospital Ulm, Ulm Germany [20]. Prior to the breast biopsies, an informed consent was obtained in a written format (under approved internal review board protocol 130/2003; University of Ulm). Lysates of breast cancer tissue and normal tissue were prepared and diluted to 200 µg/ml with assay diluent according to the manufacturer's instructions.

Before starting the assay, the capillaries and pumps were rinsed thoroughly to remove any remaining sample or beads with 20 µL 70% isopropanol (Roth, Karlsruhe, Germany). 1 µL diluted antibody-coupled beads, 1,000 for each analyte, were pumped into each capillary at a flow rate of 0.2 µl/min. The prepared tissue lysates (200 µg/ml) were then pumped into the capillary and incubated with the mixed antibody-coupled beads for 1 h at a flow rate of 0.016 µL/min. Subsequently, the mixed biotinylated detection antibodies (1X, provided in the kit) and Strepavidin-Phycoerythrin (1X, provided in the kit) were pumped into the capillary and incubated with the analytes captured on the beads for 1 h and 45 min, respectively. After the assay was finished, the beads were pumped out of the capillary in a volume of 5 µL and diluted into 100 µL with assay diluent (1X). Finally, the diluted beads were transferred into a 96-well ELISA microplate (Greiner bio-one Ltd., Frickenhausen, Germany). Followed by incubation for 5 min on a thermomixer (Eppendorf Inc., Hamburg, Germany) at 650 rpm to mix the beads in solution, the resulting beads were submitted to a Luminex 100 IS system for fluorescent signal readout (DD gate: 7,500–15,000; sample size: 80 µL; 100 events per bead region; timeout: 80 sec). The MFI value was extracted to perform data analysis.

### Normal bead-based assay

The assay was carried out according to the instructions given by the manufacturer for the WideScreen™ Receptor Tyrosine Kinase Assay Kit with minor modifications. In short, the MultiScreen HTS™ 96-well filter plate (Millipore, Inc., Massachusetts, USA) was pre-wetted with assay diluent for 5 min, 100 µL/well. Then, 50 µl diluted (1X) capture beads were transferred to each well. After removing the excess solution by filtration, 100 µL of the same tissue lysates (200 µg/ml) were added to each well to incubate with the beads at 23°C for 1 h on a thermomixer (650 rpm). Subsequently, the beads were washed 3 times with 100 µL washing buffer in each well. Then, 30 µL mixed detection antibodies (1X), 60 µL assay diluent, 30 µL streptavidin-phycoerythrin (1X), 60 µL assay diluent were transferred to each well and incubated at 23°C for 1 h and 45 min on a thermomixer at 650 rpm, respectively. Followed by 3 washing steps, 120 µL assay diluent (1X) were transferred to each well and incubated for 5 min on a thermomixer (650 rpm). After these procedures, the beads were submitted to a Luminex 100 IS system for fluorescent signal readout (DD gate: 7500–15000; sample size: 50 µl; 100 events per bead region; timeout: 80 sec).

The graphs were drawn with Excel 2003 (Microsoft Corp. Redmond, Washington, USA) and origin 7.0 (OriginLab Corp., Northampton, MA USA). The data of figure 2C,D were fit using a 4-parameter logistic fit from origin 7.0. The data of figure 3 were fit using 5-parameter non-linear fit from Xfit4.0 (ID Business Solutions Ltd. Guildford, Surrey, UK).
